# First Evidence of Feeding-Induced RNAi in Banana Weevil via Exogenous Application of dsRNA

**DOI:** 10.3390/insects13010040

**Published:** 2021-12-29

**Authors:** Henry Shaykins Mwaka, Olivier Christiaens, Priver Namanya Bwesigye, Jerome Kubiriba, Wilberforce Kateera Tushemereirwe, Godelieve Gheysen, Guy Smagghe

**Affiliations:** 1 Laboratory of Agrozoology, Department of Plants and Crops, Ghent University, 9000 Ghent, Belgium; henry.mwaka@ugent.be (H.S.M.); olivier.christiaensac@gmail.com (O.C.); 2 Department of Biotechnology, Ghent University, 9000 Ghent, Belgium; godelieve.gheysen@ugent.be; 3 National Agricultural Research Laboratories, Kawanda, Kampala P.O. Box 7065, Uganda; bwesigyep@gmail.com (P.N.B.); jkubiriba2012@gmail.com (J.K.); tkwilberforce@gmail.com (W.K.T.)

**Keywords:** RNA interference (RNAi), *Cosmopolites sordidus*, banana weevil, artificial diet, pest control, dsRNA feeding

## Abstract

**Simple Summary:**

RNA interference (RNAi), a conserved mechanism in eukaryotic organisms, is initiated by the presence of double-stranded RNA (dsRNA) in the cells, regulating the expression of specific genes by degradation of their mRNA, and it is known as post-transcriptional gene-silencing (PTGS). RNAi can be employed to develop species-specific biopesticides where dsRNA is delivered to a pest insect via the oral route. Banana weevil (*Cosmopolites sordidus*) is the most devastating pest of banana and plantain worldwide, yet current control measures are neither effective, sustainable, nor environmentally sound. This study reports an artificial diet-based multi-well plate method and the first findings on the potential application of biotechnology in the control of the banana weevil using dietary RNAi, which would arguably provide the most sustainable and practical method for dsRNA delivery. A selection of target genes with effective RNAi leading to high and rapid mortality is presented. These results indicate the potential of RNAi-mediated management to suppress *C. sordidus* efficiently in the banana crop.

**Abstract:**

Banana weevil (*Cosmopolites sordidus*) is the most devastating pest of banana and plantain worldwide, yet current control measures are neither effective, sustainable, nor environmentally sound, and no resistant farmer-preferred cultivars are known to date. In this paper, we examined the ability to induce RNA interference (RNAi) in the banana weevil via feeding. We first developed an agar- and banana corm (rhizome) flour-based artificial diet in a multi-well plate setup that allowed the banana weevils to complete their life cycle from egg through the larval instars to the pupal stage in an average period of 53 days. Adults emerged about 20 days later. The artificial diet allowed the tunneling and burrowing habits of the larvae and successful metamorphosis up to adult eclosion. Adding dsRNA for *laccase2* to the artificial diet resulted in albino phenotypes, confirming gene-silencing. Finally, *C. sordidus* was fed with dsRNA against a selection of essential target genes: *snf7*, *rps13*, *mad1*, *vha-a*, *vha-d*, and *lgl* for a period of 45 days. 100% mortality within 9–16 days was realized with dssnf7, dsrps13, and dsmad1 at 200 ng/mL artificial diet, and this corresponded to a strong reduction in gene expression. Feeding the dsRNA targeting the two *vha* genes resulted in 100% mortality after about 3–4 weeks, while treatment with ds*lgl* resulted in no mortality above the ds*gfp*-control and the water-control. Our results have implications for the development of RNAi approaches for managing important crop pests, in that banana weevils can be controlled based on the silencing of essential target genes as *snf7, rps13*, and *mad1*. They also highlight the need for research into the development of RNAi for banana protection, eventually the engineering of host-induced gene-silencing (HIGS) cultivars, given the high RNAi efficacy and its species-specific mode of action, adding the RNAi approach to the armory of integrated pest management (IPM).

## 1. Introduction

The banana weevil, *Cosmopolites sordidus* (Germar, 1824) (Coleoptera: Curculionidae) is the most important pest of banana, plantain and ensete [1]. The adult weevil is a black, hard-shelled beetle, which measures 10–15 mm, with wings seldomly used to fly. It mainly lives at the base of the banana plant between leaf sheaths, away from direct light, in a high humidity microclimate. Dissemination is primarily through infested planting material. Most adults live 1 year, while some survive up to 4 years. The female places its white, oval eggs singly into holes made by the rostrum. Most oviposition is in the leaf sheaths and rhizome surface. The rhizome, commonly known as a corm, is differentiated into a central cylinder containing the vascular bundles and an outer cortex comprised of parenchyma cells [2]. The emerging larvae preferentially feed in the rhizome. After passing through 5–8 instars, pupation is in naked cells near the surface of the host plant. Under tropical conditions, the egg to adult period is 5–7 weeks. Egg development does not occur below 12 °C; this threshold may explain why the weevil is rarely encountered above 1600 m above sea level (masl). The larvae cause extensive damage when they bore in the corm to feed and will create several tunnels through the corm and eventually the pseudostem. The tunneling causes necrosis and damage to the vascular bundles in the corm, which impairs uptake of water and nutrients, resulting in yellowing, wilting, stunting, and death of the plant [3,4]. East African highland bananas (EAHB) are highly susceptible to this pest insect, and yield loss studies have demonstrated that the banana weevil can cause up to 50% loss after the third ratoon cycle if new plantations are infested, with mat disappearance, which was previously implicated in the decline and disappearance of highland banana from its traditional growing areas in central Uganda [5,6]. Lack of effective weevil control predisposes highly susceptible bananas to possible extinction as almost happened when *Gros Michel* was replaced by *Cavendish* in Latin America [7]. With several production challenges, weevils pose an increasing concern for food security and might enhance the extinction of important bananas owing to the limited gene pool [8,9]. Presently, no single approach provides total control of banana weevils through cultural methods, while the use of natural enemies, host plant resistance, and pesticides have been shown to offer various levels of success in the control of the pest, which has encouraged the exploration of integrated pest management (IPM) strategy. IPM can be defined as a decision-based process that leverages multiple tactics to optimize and control all classes of pests in a sustainable way with the goal of reducing pesticide use, maximizing economic savings to the farmer as well as protecting the environment and human health [10,11]. Cultural methods of control include the use of clean planting material [12], hot water treatment [3], weevil traps [13], and field sanitation [14]. While there have been great successes in the use of natural enemies to control other weevils [15,16], there are limits to the use with banana weevils [17], which were described as poor candidates for biological control [18]. Although success was reported in Cuba using predatory ants *Tetramorium guineense* [19] and *Pheidole megacephala* [20], this was not achieved elsewhere [1,21]. Use of entomopathogenic fungi such as *Beauveria bassiana* and *Metarhizium anisopliae* were reported [22], but success requires high inoculum levels [23] and this is consequently not practical under field conditions. Chemical control was once an option but organochlorines, organophosphates, and other pesticides were banned in several countries [24,25] because of persistence in the environment, associated health risks [26] and pesticide resistance [27]. The resistance to pesticides, as evidenced by a large pool of detoxification enzymes in the midgut [28], further necessitates a different strategy.

The various attempts to control banana weevils using cultural, chemical, and biological methods [17] have not been very effective because they are labor-intensive [13], expensive, or not environmentally friendly [29], and this has remained a substantial problem for millions of farmers in Africa. Therefore, the best IPM strategy under the circumstances would be the use of host plant resistance in combination with cultural control methods highlighting the need for resistant cultivars. Breeding for resistant varieties would be a means to address the pest problem, but there are no resistant farmer-preferred cultivars known to date [30]. Hence, there is a need to explore reported successes in the use of RNA interference (RNAi) via the plant [31].

RNAi is a natural, conserved biological process through which double-stranded RNA (dsRNA) regulates the expression of specific genes by degradation of their mRNA into small interfering RNAs (siRNAs), and it is known as post-transcriptional gene silencing (PTGS). The RNAi mechanism was described in plants as an antiviral defense process [32], but it is also functional in many different insect species [33]. RNAi as a sequence-specific process fits in the search for an environmentally friendly strategy to control important pest insects such as the banana weevil considering that the use of pesticides is about 2 million tons annually [34], yet they are very toxic in nature and pose acute risks on the human health and the environment [35]. Studies have shown a half-life of less than 24 h for dsRNA within the soil, primarily due to bacterial degradation [36], which presents an opportunity to mitigate risks caused by the ever-increasing need for the use of pesticides. An important landmark was the development of maize resistant to the western corn rootworm (WCR) by the expression of insect-specific dsRNAs in maize plants, and this application is now accepted for commercialization in northern America (USA and Canada) and also in southern America in Brazil [37,38]. In bananas, intron hairpin RNA (ihpRNA)-mediated expression of small interfering RNAs (siRNAs) targeting vital fungal genes resulted in durable resistance against Fusarium Wilt [39]. Additionally, findings on the successful development of transgenic plants expressing dsRNA with ihpRNA constructs against the banana bunchy top virus (BBTV) [40] provided compelling evidence for exploration of RNAi in the control of banana weevil.

Lately, there have been reports on the potential for use of RNAi against weevils [28,41,42,43,44,45], which generated the need for further research. In one study, the insecticidal potency of dsRNA against weevils through retarded growth and mortality of larvae following treatment of eggs with dsRNA was reported [44]. Considering that weevil eggs are laid in shallow cavities, surrounded by sap and necrotic tissue [46,47,48], also a microbe-rich environment, which enhances rapid degradation of dsRNA by microbial ribonucleases [36,49,50], there is limited scope for host-delivered dsRNA targeting the egg stage. A more viable approach was focusing on dsRNA delivery to the destructive feeding stage of the banana weevil. The larvae would provide useful insights on host-delivered dsRNA applications. In this study, we sought to know if feeding the larvae of the banana weevil with dsRNA could also induce an RNAi response and, in turn, kill the insects. We first needed to develop an artificial diet-based methodology that allowed the banana weevil larvae to complete their life cycle up to the pupal and adult stage. We used a recipe based on agar and banana corm (rhizome) flour [51] and prepared this diet in a multi-well plate setup. In the second series of experiments, the banana weevil was tested for its sensitivity to RNAi for which dslaccase2 was chosen to feed the larvae. Based on previous experiments in the African sweet potato weevil, silencing of this *laccase2* gene results in albino phenotypes [52]. After confirmation of RNAi in *C*. *sordidus* using the albino phenotype caused by dslaccase2, the artificial diet-delivery method was used to investigate a selection of essential genes for their potential to control the banana weevil by oral RNAi. Essential genes as *snf7*, *rps13*, *mad1*, *vha-a*, *vha-d,* and *lgl* were chosen as target genes. DsRNA design was conducted using a bioinformatics pipeline for biosafety to humans and the environment. Gene-specific dsRNAs were prepared in vitro, incorporated into the artificial diet, and then fed to banana weevils. Here, we used control groups (dsgfp- and water) to discriminate mortality. The gene-silencing effect at RNA level for the 3 most active dsRNAs (dssnf7, dsrps13, and dsmad1) was confirmed by comparing entomotoxicity to the reduced expression level of the target gene.

## 2. Materials and Methods

### 2.1. Collection of Banana Weevils and Maintenance on Banana Pseudostems

Banana weevils (*C. sordidus*) were collected on 25 September 2014 from the Banana Germplasm collection hosted at the National Agricultural Research Laboratories Institute in Kawanda. Kawanda is located 13 km northern of the city of Kampala, Uganda, at an elevation of 1195 masl (0°25′N, 32°32′ E). Average daily temperatures were 15 °C minimum and 29 °C maximum with a mean relative humidity of 76%. Kawanda receives a mean annual rainfall of about 119 mm per year in a bimodal distribution. The weevils were raised on fresh corms and provided with pseudostems, which is preferred for oviposition by the egg-laying females. Weevil traps were made from freshly dissected pseudostems of about 30 cm long, which were laid beside flowering *Mbwazirume* East African Highland Banana plants. After 3 days, weevils were collected from the traps and reared on detached corms (*Mbwazirume*) in 20 liter-buckets. Sex determination of the weevils was carried out using a method described previously [13,53] by viewing punctuations on the rostrum with a stereomicroscope at ×10 magnification. The weevils were separated into 2 containers based on sex. A total of 600 weevils on corm tissue were transported to Belgium on 30 October 2014 and again in 2015 using permits of the Federal Agency for the Safety of the Food Chain (FAVV, Brussels, Belgium).

In Belgium, the weevils were taken to the Laboratory of Agrozoology at Ghent University, followed by acclimatization to the new environment for a period of 3 weeks. The banana weevils were maintained on pseudostem tissue and leaves of banana cultivar *Silver bluggoe* (obtained from ILVO, Merelbeke, Belgium) until a suitable artificial diet was available 6 weeks later.

### 2.2. Preparation of Artificial Diet

Artificial diet was prepared based on a formulation for sweet potato weevil [54] with small adaptations: sweet potato was replaced by banana corm powder, tetracycline was excluded, and dextrose was reduced from 40 g/L [51] to 10 g/L. In summary, banana corm was extracted from 6-month-old potted plants of *Silver bluggoe* (obtained from ILVO, Merelbeke, Belgium), washed, thinly sliced, and dried on a tray in an oven at 40 °C for 24 h. Dried slices were ground to a fine powder and stored in several 50 mL-falcon tubes at room temperature.

As shown in Table 1, 1 L of the diet was prepared with 80 g corm powder, 10 g dextrose, 14.4 g cellulose, 21.6 g casein, 9.0 g yeast extract, 2.7 g Wesson’s salt mixture, 0.45 g choline chloride into a 2 L-flask (on a magnetic stirrer) with 500 mL of distilled water containing 675 mg of dissolved Nipagin (Sigma-Aldrich, Saint-Louis, MO, USA). When all the components had dissolved, another 330 mL of distilled water was added and further stirred for 10 min prior to pH adjustment to 7.0. Finally, 20 g agar was added, and the mixture was heated with continuous stirring until boiling into a smooth porridge mixture. This was allowed to cool to about 55 °C at which point 10 mL of 100% ethanol containing 0.36 g inositol, 0.7 g stigmasterol, 0.675 g potassium sorbate was added, followed by the addition of 10 mL of a filter-sterilized solution of 0.045 g B-vitamin mixture and 1.8 g ascorbic acid processed through a 0.22 μm-filter. The volume was made up to the 1 L-mark of the graduated flask with distilled water. The heat was maintained with stirring at 55 °C until all the artificial diet was dispensed into appropriate culture vessels and storage containers.

### 2.3. Assessing Development of Weevil Stages on Artificial Diet

About 300 female banana weevils were introduced on a freshly cut pseudostem in a transparent plastic container for oviposition. After 3 days, the eggs were gently pried out of the pseudostem with a knife and set in a Petri dish (9 cm diameter) with sterile water for decontamination. Batches of 100 eggs were rinsed 3 times with sterile water and once with a 2% hypochlorite solution, and then laid onto a moistened piece of tissue in a Petri dish, incubated at 25 °C and moistened daily with a water spray until hatching. Where no contamination occurred, the red-headed larvae emerged from the eggs within 7–10 days. Neonates were immediately compartmentalized on artificial diet plates to prevent cannibalism [2].

To evaluate the suitability of the diet for the egg stage, the experimental setup comprised a simple completely randomized design (CRD) of 3 replicates with 24 eggs randomly placed on the surface of the artificial diet in a 9 cm-Petri dish and observed for 10 days in comparison to a similar setup for controls with 24 eggs on moist tissue in a 25 °C incubator (Panasonic MLR-352H, Oizumi, Gunma, Japan). To evaluate the suitability of the diet for the larval development stage, a toothpick was used to transfer neonate larvae to the surface of artificial diet in a 9 cm-Petri dish, and this was conducted with 3 biological repetitions, each consisting of 20 neonate larvae. After 4 days, the larvae were separated into 24-well plates for better observation. They were monitored for survival and weighed every 4 days until pupation for those that survived. To determine fresh weight, the artificial diet at the top of the well plate was carefully removed with a toothpick to expose the burrowed larva. The exposed larvae were picked up with a toothpick or forceps depending on age/size and placed into a clean and dry weighing boat, which was then placed on a weighing balance to determine weight. Once weighed, the larvae were returned to the well. The data on weight were analyzed with ANOVA followed by a Fisher LSD test. Complete data from 10 surviving larvae, over a period of 68 days was used to compute the growth rate. To determine the suitability of the artificial diet for adult weevils, 10 males and 10 females, that were singly placed in a modified concentric dish with access to a dish containing artificial diet, were observed over a period of 20 days. The observations from the artificial diet assay provided a method for testing the efficacy of dietary dsRNA against banana weevil.

### 2.4. Target Gene Selection and dsRNA Synthesis

The criteria for the selection of a target gene were premised on the basis of the dsRNA’s potential to produce a high and rapid toxic effect when ingested. The choice of gene targets (*snf7, rps13, mad1, vha-a, vha-d,* and *lgl*) selected for this study was based on findings from previous studies with other insects where mortality was attributed to the dsRNA administered either through injection or feeding [28,52,57,58,59]. The banana weevil orthologs were identified with TBLASTN using *Tribolium castaneum* and *Cylas puncticollis* protein queries against weevil transcriptome data (privately shared by Venganza Inc., Raleigh, NC, USA) (Table S1). Using primers, the genes were amplified, cloned, and confirmed by sequencing as described below. For the selected gene segments, lengths greater than 60 base-pairs (bp) were selected as this was reported to be required for biological activity in artificial diet assays [60,61]. *Laccase2* was the first candidate gene selected because it presents an easily assayable phenotype providing a visual method to evaluate a gene-specific RNAi response of various insects. The *laccase2* gene was shown to be responsible for cuticle tanning in *Tribolium castaneum* [62] and *Cylas puncticollis* [52]. *Snf7* encodes a subunit protein of the ESCRT-III complex (Endosomal Sorting Complex Required for Transport), which plays a housekeeping role in the internalization, transport, sorting, and lysosomal degradation of transmembrane proteins. It was chosen because it was an essential gene, and silencing of *snf7* in WCR through feeding resulted in stunting and death [59]. The *rps13* gene encodes the ribosomal protein S13 that is a component of the 40S subunit of ribosomes and plays an important role in protein synthesis as a structural component in ribosome assembly. It was selected because gene-silencing studies had demonstrated its potential to cause mortality [58,63,64], when dsRNA was ingested. *Mad1* was selected as a target because mutations in *mad1* were documented to be lethal owing to the failure of the spindle assembly checkpoint, a process which is necessary to ensure correct chromosome segregation by deferring commencement of anaphase before all sister chromatids are correctly aligned [65,66]. RNAi of *mad1* was evaluated against the sweet potato weevil, where it caused mortality [52]. Vacuolar-type ATPase (vha-a and vha-d) was the catalytic subunit of a multisubunit protein responsible for ATP hydrolysis [67] and was selected as a suitable target because its silencing resulted in mortality and reproduction failure in the cotton bollworm [68]. The large giant lethal protein gene (*lgl*) was selected following reports that 100% mortality was attained within 20 days following the oral administration of dsRNA against *lgl* to *T. castaneum* larvae [69]. Lgl is a conserved membrane-associated scaffold that functions in concert with Scrib and Dlg scaffolds and apical Par6/aPKC complexes to establish apical-basal cell polarity, cell proliferation, differentiation, and tissue organization. Target regions from selected genes were chosen after analysis to identify regions with a higher probability of generating potent small interfering RNAs (siRNAs). Briefly, selected gene sequences were analyzed in-house with software hosted at the Whitehead siRNA Selection Web Server [70] to assess a region with the highest potential for generating potent siRNAs and identify sections with no off-target hits using the human and mouse dbs. Suitable regions were further queried against NCBI databases and assessed for hits against other insects with bees included.

In all tests, total RNA was isolated from 10-day-old banana weevil larvae, using the RNeasy mini kit (Qiagen, Venlo, The Netherlands) followed by digestion with RQ1 RNase-Free DNase kit (Promega, Madison, WI, USA) according to manufacturer’s specifications to remove any remaining DNA from the RNA samples. First-strand cDNA synthesis was carried out using oligo-dT primers. PCR was carried out to amplify selected regions of the different target genes using a Bio-Rad Thermocycler with the primers listed in Table S2. PCR conditions were 94 °C for 5 min, followed by 35 cycles of 94 °C for 30 s, 55 °C (for all gene targets) for 30 s and 72 °C for 45 s, finishing with an extension step at 72 °C for 10 min. Then, the amplified PCR products of the targets were first cloned in pGEM®-T Easy vector (Promega, Madison, WI, USA) and then subcloned in between the bi-directional T7 promoter of the Litmus38i vectors (New England Biolabs, Ipswich, MA, USA). As illustrated with *laccase2*, the fragment was subcloned using restriction enzymes (*Apa*1 and *Sal*1) (Figure S1) into the *Escherichia coli* strain HT115(DE3), which is deficient in RNase III [21,22]. To produce dsRNA, the recombinant plasmid was transformed into *E. coli* HT115(DE3) strain and colonies containing transformants were screened by PCR, and then the produced dsRNA was purified from the HT115 DE3 *E. coli* strain, as previously described [71]. Briefly, the *E. coli* HT115 containing the recombinant Litmus38i with various gene targets were cultured in 4 mL of LB (10 g/L tryptone, 10 g/L yeast extract, 5 g/L NaCl at pH 7.0) broth medium amended with carbenicillin (100 μg/mL) and tetracycline (12.5 μg/mL) at 37 °C with shaking at 200 rpm overnight. This was followed by inoculating 25 mL of LB with a 250 µL-aliquot of the preculture amended with the same antibiotics as above in a 125 mL-conical flask. The culture was incubated at 37 °C while shaking at 200 rpm until the culture attained the suitable OD600 of 0.4 (about 3.5 h later). To activate the T7 promoter for RNA transcription, IPTG was added at 1 mM concentration and incubation conditions were maintained for 4 additional hours. The cells were harvested by centrifugation at 7000 rpm for 10 min and resuspended in 8 mL of physiological solution. One mL-aliquots were centrifuged at 7000 rpm for 10 min in 1.5 mL-tubes and stored at −80 °C. Extraction and purification of dsRNA from the bacterial cells involved cell lysis with TES buffer (10 mM Tris pH 7.5, 10 mM EDTA and 0.5% SDS), ssRNA was removed by incubation with 5 µL of RNase A (1000 U/µL) in 10× RNase A buffer (4 M NaCl, 0.1 M Tris-HCl) while dsRNA was purified using TRI reagent (Sigma-Aldrich) followed by extractions with chloroform and ethanol as previously described [72]. To quantify dsRNA obtained, actual absorbance was determined with a Nanodrop 2000 UV visible spectrophotometer (Thermo Fisher Scientific, Waltham, MA, USA) and concentration was estimated using a conversion factor of 46.74, as previously described [73].

### 2.5. Bioassay with dsRNAs with Banana Weevils on Artificial Diet

Prior to the commencement of the main study, a preliminary experiment was performed to optimize handling techniques and determine optimum concentrations for assessment of mortality resulting from dietary dsRNA. For each dsRNA concentration, 8 neonate larvae were evaluated over a period of 8 days, and 6 concentrations of 0, 50, 100, 200, 400, and 800 ng/mL of diet were used. This experiment was repeated twice, and the data obtained was used to setup the main study as described below.

The optimized setup for growth and evaluation of banana weevil larvae on an artificial diet involved seeding in 96-well plates with a single larva per well immediately after hatching and followed by 2 transfers to 24-well plates, a 1st time on day 8 and a 2nd time on day 29. Briefly, 200 µL of the diet was dispensed into each well of a 96-well plate and allowed to cool and solidify for 3 h in a laminar flow hood. Each well was seeded with a single larva; the plate was wrapped with aluminum foil and set in a 25 °C incubator (Panasonic, Osaka, Japan). The plates were observed daily to ensure no contamination and to check the survival of the larvae. An additional 100 mg of diet was added every 3rd day to ensure availability of sufficient food and a suitable environment to support the burrowing habits of the banana weevil larvae. In the event of suspected contamination or death, the well was carefully excavated, contents disposed of, and the well was cleaned with 70% ethanol, followed by rinsing with distilled water. On the 8th day, the living larvae were transferred to a 24-well plate, and daily monitoring was continued.

The first part of the bioassay involved a study with dsRNA targeting the *laccase2* gene to assess the effect on cuticle tanning in the banana weevil larvae. Hereto, 2-days-old larvae were used and kept for 45 days on an artificial diet with 800 ng/mL of dslaccase2 or control dsRNA in multi-well plates as described above. The individuals were followed for survival and morphological abnormalities such as albino phenotypes up to adult formation.

To assess the effect of dietary dsRNA on survival over a period of 45 days, the experiment was setup in a CRD using an artificial diet with 200 ng of dsRNA per mL of diet for *snf7, rps13, vha-a, mad1, vha-d,* and *lgl*, and with dsgfp and water as controls. The insects were kept on artificial diet as described above for a period of 45 days. Larvae were placed into the wells and each treatment was performed with 30 biological replicates and repeated twice. The plates were wrapped in aluminum foil, and each well was individually observed daily to determine the living status of the larvae. Data were collected daily from a total of 270 wells for up to 45 days. Binary data comprising of 1 and 0 were collected to represent the living status. A “0” showed that the larva was dead, while a “1” indicated that the larva was alive. Death was confirmed by the excavation of the well and observation of the insect larva. Life was confirmed by visual observation of movement within the well. For insect larvae that had burrowed, thin layers of the diet were carefully removed until the larva was exposed for an accurate assessment of survival to be made. The cumulative number of larvae that died was used to calculate the percentage of mortality, and these data were analyzed with ANOVA followed by a post-hoc Tukey test. The graphs were generated, and ET_50_ values (i.e., the time needed to realize 50% of mortality of treated insects) together with the 95% confidence interval were calculated by sigmoid curve fitting in Prism V8.4 (GraphPad, San Diego, CA, USA).

### 2.6. Semi-Quantitative PCR Analysis of Gene-Silencing Effect with dsRNAs

To link entomotoxicity with gene-silencing, we performed an extra experiment with semi-quantitative PCR analysis of the expression of the target genes after larvae were treated with gene-specific dsRNAs (800 ng/mL) against the target genes *snf7, rps13, mad1* or with the dsgfp control for a period of 48 h on artificial diet. These conditions were chosen as these were expected to cause gene-silencing before the insects are dead, as in agreement with previous experiments [52,58]. In brief, larvae of 28 days old were treated as described above in the insect bioassay on an artificial diet in a 24-well plate, except for the use of a higher concentration dsRNA to compensate for the shorter treatment period and the larger size of the larvae. After the treatment, the larvae were collected individually, frozen in liquid nitrogen, and stored at −80 °C. RNA extraction was performed using TRIzol reagent (Invitrogen, Carlsbad, CA, USA), following the manufacturer’s instructions. Hereto, 500 ng of total RNA was used as a template for synthesis of the first strand of cDNA with an oligo-(dT) primer and the Maxima H Minus cDNA synthesis kit (Thermo Scientific, Hudson, NH, USA). All cDNA pools were screened in a multiplex PCR with actin and gene specific primers (Table S2). The PCR reaction was carried out in a final volume of 25 μL. PCR conditions were: 95 °C for 3 min, 60 °C for *snf7* and *rps13* and 58 °C for *mad1* during 30 s, and then finally 72 °C for 30 s for 28 cycles, followed by 5 min of extension at 72 °C. All PCR products were resolved by electrophoresis on 1% agarose gels, and the images were analyzed by ImageJ.

### 2.7. Statistical Analysis of Results

All statistical analyses were performed using Minitab 19 Statistical Software (State College, PA, USA), while the survival curves and graphs were drawn by GraphPad Prism 8 (GraphPad, San Diego, CA, USA) and Microsoft Excel.

## 3. Results

### 3.1. Banana Weevil Development on Artificial Diet in Multi-Well Plates

At first, we investigated the success of the artificial diet with different developmental stages as eggs, neonate larvae, and adults. As shown in Figure S2A, when placing freshly laid eggs that were collected from the banana pseudostem leaf sheaths on the artificial diet, most (72%) of these did not hatch within the 10 day-observation period. These eggs turned pale and some brown, and also, by the 10th day, there was no indication of development for the eggs under the binocular. In contrast, when eggs were incubated on moist paper tissue under the same conditions, 79% hatched by the 10th day, and larvae were active, as shown in Figure S2B,C by the energetic movement across the surface of the tissue. The larvae, when left on this tissue, appeared to aggregate close to each other and were cannibalistic. Finally, based on these earlier observations, we realized an improvement of larval survival on the artificial diet by keeping the eggs for 6 days on the moist tissue paper and then transferring these to the artificial diet. The hatched larvae were normal and appeared cream-colored, curvy, with the characteristic reddish-brown head mandibles that readily punctured the artificial diet into which they quickly burrowed (Figure S3C,D).

**Figure 1:**
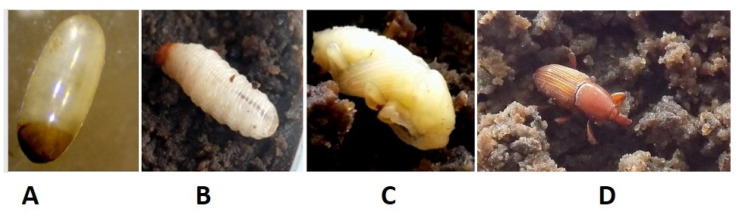

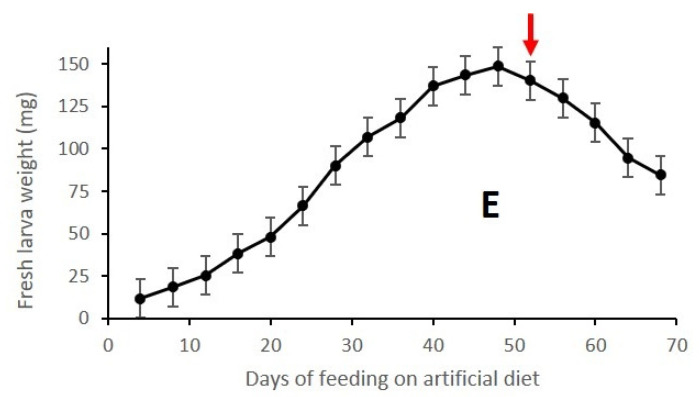
The developmental stages of banana weevil on artificial diet: (**A**) egg, (**B**) larval stage, (**C**) pupa, and (**D**) adult. Typical is the increase in dark color after adult eclosion: young individuals with light brown color. (**E**) Fresh weight of the larval stages (in mg) over the 70 days of feeding on an artificial diet. The red arrow indicates that the larvae developed into pupae at day 53.

When neonate larvae were placed on the surface of the artificial diet in 96-well plates with one larva per well, they readily began to feed on the diet as seen by bite marks and general disturbance on the surface of the diet (Figure S3E). Within a period of 3 h, all larvae had burrowed in and tunneled through the diet. On day 8, the larvae were transferred the first time to a new artificial diet in a 24-well plate with one larva per well. Later on, the larvae were transferred a second time to a new diet at day 29 in a new 24-well plate with one larva per well (Figure S3E,F). In this experiment, the larvae were followed until pupation and weighed every 4 days, and a one-way ANOVA followed by a post-hoc Fisher test was performed (Table S3A–C). The average larval weight on the 4th day was 11.7 mg (±1.8), increased to 25.3 mg (±3.6) on day 12 and 66 mg (±3.4) on day 24, and peaked at 148 mg (±4.7) by day 48 (Figure 1).

The average time from egg to pupation (Figure 1C,E) was 53 days (±4.7). At the event of pupation, the larvae became less active and burrowed into enlarged tunnels close to the surface of the diet. The pupation process occurred inside the diet in pods that were created by the larvae, and observation required excavation. The pupal stage took, on average, 20 days (±2.5), and they gradually turned brown.

When 20 adults, comprising 10 males and 10 females, were placed on the artificial diet, the weevils migrated towards the diet and were observed feeding within a couple of minutes; both males and females responded to the diet. Typically, the weevils burrowed in the diet, as shown in Figure 1D and Figure S2F–H.

### 3.2. Banana Weevils Are Sensitive for Oral RNAi with dslaccase2, Resulting in Albino Phenotype

**Figure 2:**
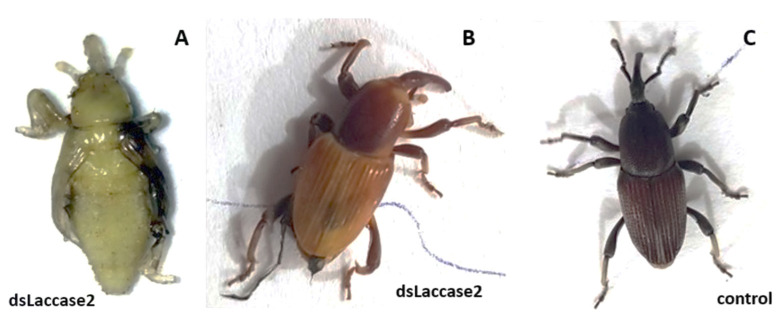
Feeding on an artificial diet with dslaccase2 (800 ng/mL) caused albino phenotypes (**A**,**B**) in the adult stage as shown, while the control (**C**) had a hard sclerotized black body.

Using the artificial diet setup with neonate larvae as presented above, we investigated the sensitivity of banana weevils for oral RNAi, and we hereto used dslaccase2 as previous in-house experiments in different insects demonstrated albino individuals by specific gene-silencing. With the larvae that were fed on dslaccase2 at 800 ng/mL diet, there was low mortality during the experiment, comparable to the controls. Further on, 71% of the larvae had pupated by day 55, and these were observed as white oval-shaped individuals with developing appendages (Figure 2A). All these dslaccase2-treated pupae were white albino, and they were alive as indicated by the tactile response of the distal abdominal part when touched. Further on, 29% of the pupae had successfully developed into the adult stage, and the resulting adults had albinism (Figure 2B). In the control group, 79% of the larvae had pupated by day 55, and all showed normal morphology, and further on, 89% of these pupae had eclosed as a normal adult at day 66, representing 70% survival in total.

### 3.3. Target Gene Selection with High and Rapid Mortality by Oral RNAi

In preliminary experiments, different concentrations of dsRNA for the target genes were tested in the artificial diet (data not shown), and 200 ng/mL was chosen for further research. As an illustration, with dssnf7 at 50, 100, 200, 400, and 800 ng/mL, we scored average mortality of 21, 32, 36, 61, and 74%, respectively.

Then with the main optimized experiment where all dsRNAs were dosed at 200 ng per mL in the diet, a one-way ANOVA was conducted to compare the effect of different dsRNA on mortality, as shown in Table S4. There was a significant effect of dsRNA on mortality at the *p* < 0.001 level. A level of *p* < 0.05 by a post-hoc Tukey test was accepted as statistically significant, and Table S4E provides groupings where means followed by the same letter in the same column are not significantly different. The strongest mortality was obtained with dssnf7 and dsrps13, followed by a group of dsmad1, then followed by a group with dsvha-a and dsvha-d, and finally dslgl was not toxic, similar to the water control.

**Figure 3:**
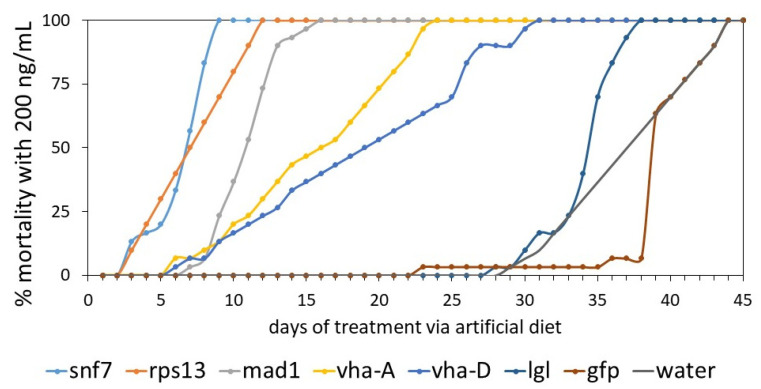
Mortality graphs when banana weevil was fed with dsRNA via artificial diet. A total of 30 neonate larvae were placed on the diet that was supplemented with 200 ng of dsRNA per mL of diet. The mean survival of 30 larvae per dsRNA treatment was calculated. The graphic is a representative of the mortality by dssnf7, dsrps13, dsmad1, dsvha-a, dsvha-d, dslgl, and compared to the dsgfp-control and water-control over the 45-days period of the experiment; data were analyzed by ANOVA and Tukey (Table S4). In the water-control, no mortality was observed for 29 days; the survival was similar to the dsgfp-controls (Figure S4). The Kaplan-Meier statistical analysis in GraphPad Prism v8.4 (GraphPad, San Diego, CA, USA) was used for comparing the mortality of different dsRNA with the dsgfp (see Table S6).

Figure 3 presents the mortality curves over time of the experiment for the different dsRNA tested at 200 ng/mL in the artificial diet. It is clear that dssnf7, dsrsp13, and dsmad1 showed 100% mortality and this was within 1–2 weeks. In detail, 56% mortality was observed with dssnf7 at day 7 and 100% at day 9. When compared with the dsgfp-control (Figure S4), the two mortality curves fitted on the same horizontal axis were at the widest separation for the dsRNA evaluations in this study. For dsrps13, there was 30%, 80%, and 100% mortality at days 5, 10, and 13, respectively (Table S6), while for dsmad1 37% and 100% mortality was realized after 10 and 16 days, respectively. The mortality curve (Figure S4F) comparing the mortality arising from dsrps13 with the dsgfp-control shows a strong entomotoxic effect associated with dsrps13 similar to dssnf7 as shown with ANOVA (Table S4). Compared to dssnf7 and dsrps13, dsmad1 also showed a strong entomotoxic effect when compared with the dsgfp-control with 37%, 73%, and 100% mortality on days 10, 12, and 16, respectively (Table S6). For the other dsRNAs tested, the entomotoxic effect was lower. For instance, with dsRNA against the two *vha* genes, feeding resulted in 100% mortality after about 3–4 weeks, while dslgl showed little mortality above the dsgfp-control (Figure S4) and the water-control. In detail, with dsvha-a and dsvha-d, respective mortalities of 30% and 23% were realized at day 12, and these were 73% and 53% at day 20. 100% mortality was achieved on day 24 for dsvha-a and day 31 for dsvha-d (Table S6).

As shown in Table S5, the respective ET_50_ values for 200 ng/mL of dssnf7, dsrps13 and dsmad1 were 6.4 days (5.8–7.0), 6.6 days (6.1–7.1), and 10.6 days (10.4–10.8), while this was 15.1 days (14.5–15.7) for dsvha-a, 18.0 days (17.1–18.6) for dsvha-d and 34.2 days (33.9–34.3) for dslgl. The ET_50_ value was 39.2 days (38.9–39.5) in the dsgfp-control and 36.8 days (36.6–37.0) in the water control.

### 3.4. Semi-Quantitative PCR Analysis of Gene-Silencing Effect with Dietary dsRNAs

**Figure 4:**
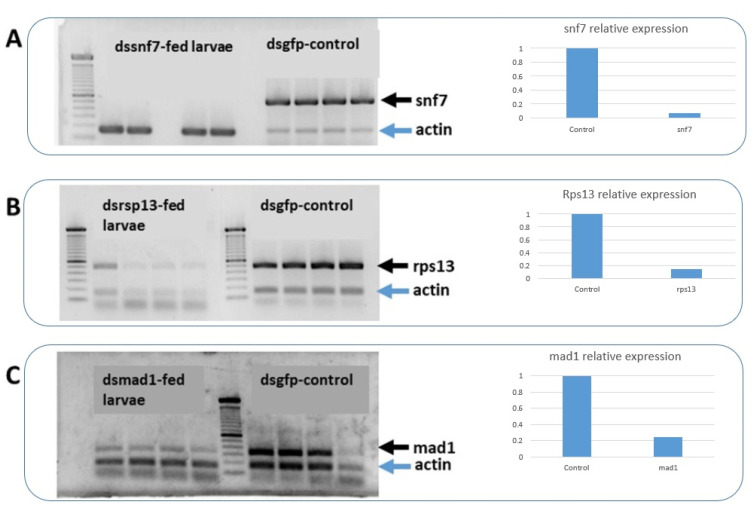
Semi-quantitative PCR to check the level of mRNA of the target genes after feeding dsRNA to banana weevil. On the left, four replicates of the treatment with the dsRNA for the target gene, and at the right four replicates of the control-treated with dsRNA for gfp. (**A**) dssnf7, (**B**) dsrps13, and (**C**) dsmad1. The right panels show the results of the ImageJ analysis.

Figure 4 demonstrates that the expression of the target genes *snf7*, *rsp13,* and *mad1* was reduced in larvae that had fed on the respective dsRNA in the artificial diet. Indeed, these data confirm that 48 h feeding is sufficient to degrade the production of the respective mRNA to levels not detectable by RT-PCR after 28 cycles. In detail, the silencing for *snf7* was total as no band was observed in the treated larvae. For dsrsp13 and dsmad1, the effect at mRNA level was somewhat lower.

## 4. Discussion

The banana weevil *C. sordidus* is the most devastating pest of banana and plantain worldwide, and it has been at the center of attention for plant breeders who have continued to pursue the development of host plant resistance as the only sustainable way to address the growing food security threat attributed to the pests [1,2,3]. While biological control has been considered, it remains ineffective [23]. Following the reports of successful RNAi in important pest insects in agriculture [37,38,57], this study was initiated to investigate the potential use of oral RNAi in the control of the banana weevil. However, prior to assessing the sensitivity of banana weevil larvae to dsRNA, it was necessary to develop a standard bioassay using an artificial diet. We choose to test the larval stages as these are the most destructive stage of the banana weevil. In this study, we optimized an artificial diet, and hereto we used corm (rhizome) powder and supplemented this with dextrose and cellulose to additionally provide carbohydrates, while the protein was mainly from casein and yeast extract as well as vitamins. The results demonstrated that this artificial diet supports the growth of weevil larvae and their development up to adult eclosion. Once the larvae were introduced to the diet, they readily consumed and burrowed into the diet and migrated normally through the tunnels that they made. The suitability of the diet was reflected in average weight of the larvae of 148 mg (±4.7) after 48 days, which is comparable to when larvae are feeding on corm tissue of EAHBs (*Atwalira*, *Kisubi*, *Mbwazirume,* and *Kayinja*) [74]. On the duration of the larval stage, this was 53 days (±4.7) in our experiments, and this is somewhat longer than 47.8 (±4.7) days reported in the moderately resistant *Kisubi* and 28.4 (±0.5) in the susceptible *Atwalira* cultivar [74]. Here it is also recommended that the assessment to evaluate effect on growth of larvae should be carried out at a time when the larvae are less prone to injury during extraction from the artificial diet. The best time for assessment would, therefore, be between 12–28 days when the growth rate is steady. The advantage is that the use of artificial diets utilizes a natural delivery method through feeding and is associated to a low concentration of host-delivered dsRNA.

The next goal of this study was to investigate the sensitivity of banana weevil larvae to RNAi. A suitable target for RNAi-based control would ideally result in a rapid toxic effect in the weevil larvae since the goal is to achieve mortality of the pest as soon as possible after it starts to feed on the host. Prior to feeding the larvae with dsRNA against a selection of essential target genes, we added dsRNA for *laccase2* to the artificial diet and this resulted in albino phenotypes, confirming gene-silencing based on previous reports in different insects [52,62]. Thus, we can conclude that this artificial diet assay not only proved the RNAi functionality in *C. sordidus*, but also that feeding dsRNA resulted in an efficient knockdown. Subsequently, we made a selection of essential target genes, meaning genes, which are expected to lead to toxicity and lethality when successfully knocked down, and this was based on literature and in-house experience. Our selection consisted of *snf7*, *rps13*, *mad1*, *vha-a*, *vha-d,* and *lgl*, and these were dosed at 200 ng/mL for a period of 45 days. Our study demonstrated that the banana weevil response varied depending on the gene targeted, with dssnf7 ingestion resulting in fast and high mortality, followed by dsrps13, dsmad1, dsvha-a, dsvha-d, and dslgl. Using dssnf7, 100% mortality was achieved by day 9 as compared to day 13 in dsrps13 and day 16 with dsmad1, while the other dsRNAs were lower in activity or not above the controls. These findings are consistent with previous reports such as in WCR (*D. virgifera virgifera*) where dssnf7 resulted in death after 5 days of exposure [60], or in the Sri Lanka weevil (*Myllocerus undecimpustulatus*) where feeding with dssnf7, dsrps13, and dsv-ATPase resulted in 93% 64%, and 43% mortality, respectively [75]. The observed ranges for the insecticide effects between dsRNAs suggest that different factors influence the potency of exogenous dsRNA such as stability of dsRNA in the diet and insufficient dsRNA internalization [76]. Indeed we cannot rule out the possibility of (side)effects induced by degraded dsRNAs or other byproducts. In addition, we acknowledge that the application of dsRNAs as a pesticide in the field may be challenged by their instability at environmental temperature and slow effect on mortality.

The high sensitivity of banana weevils to orally administered dssnf7 makes it a prime choice for future works. As demonstrated, the silencing for *snf7* was total as no band was observed in the treated larvae (Figure 4), and interestingly this agrees with the rapid and total mortality with this dsRNA (100% mortality in 9 days). For dsrsp13 and dsmad1, the effect at mRNA level was somewhat lower, which corresponds to the somewhat lower entomotoxic activity as observed in the insect bioassays. This high mortality was not surprising since prior studies demonstrated high mortality by dssnf7 also in other insects. For instance, feeding of DvSnf7 dsRNA to WCR larvae resulted in stunting and death with an LC_50_ of 4.3 ng of dsRNA per mL of diet [77]. Additionally, feeding dssnf7 in WCR was documented to elicit RNAi within 10 h and have a long-lasting effect of up to 40 days after exposure [78]. The high mortality by dssnf7 can be explained by the important role of the target gene (*snf7*), being a part of the ESCRT pathway, which functions in cellular housekeeping by internalization, transport, sorting, and lysosomal degradation of transmembrane proteins. Specifically, dssnf7 is causing the death of the insect by impairing the autophagy process, which is evident in the accumulation of ubiquitinated proteins in the larval tissues after ingestion of dssnf7 [77]. We believe that the low concentrations and high mortality with dssnf7, together with the high RNAi efficacy and its species-specific mode of action [60,79,80,81] make a case for the utilization of the approach in environmentally safe pest management solutions.

## 5. Conclusions

As a naturally occurring process in organisms, innovations harnessing RNAi in pest management offer a safer alternative with the potential to reverse the adverse effects pesticides have had on the environment. The biggest benefit with the technology is that RNAi can be tailored to a single pest, considerably reducing the risk of collateral damage that is usually associated with the use of pesticides that are generally indiscriminate. The sequence-specific mode of action of RNAi-based products makes risk assessment easier for organisms with published genomes. In comparison to transgenic plants expressing resistant proteins that affect pests, RNAi technology in product development is bound to be deregulated faster, considering that absence of novel proteins in food products is likely to reduce consumers’ fears associated with the genetic engineering of plants.

The power of RNAi in pest management can be utilized in various ways such as spraying, irrigation, injection (SIGS, spray-induced gene-silencing), and transgenic plants expressing dsRNA (HIGS, host-induced gene-silencing) [37,81]. The most cost-effective delivery method should take into account the behavior of the target pest, the economics of delivery, and the effectiveness of the method. While spraying or trunk injection may not be suitable for banana weevil larvae and the biology of the banana crop, engineering banana plants to produce highly potent dsRNAs for targets such as *snf7* would effectively deliver the payload to the pest, which feeds in the corm tissue. HIGS utilizing the plant’s machinery to produce dsRNA and siRNAs targeting pests and pathogens was explored against insects [82], nematodes, viruses [83], fungi [84], and parasitic plants [85]. The case of exploration of RNAi technology in banana in weevil pest management is strong mainly because of the biology of the banana weevil. The most destructive stage of the banana weevil, namely the larvae, live and feed within the corm, and no available control method can effectively tackle the pest from the safety of the corm. Most control methods target the adult weevil and not the weevil larvae and, therefore, do not directly stop the destruction of the plant. If the weevil is able to evade the control measures and lay eggs on the host plant, then the cycle of destruction will continue. The most effective long-term strategy is the development of host plant resistance, which would make bananas unsuitable for the proliferation of the weevil. The biggest benefit with the technology is that RNAi could theoretically be tailored to a single pest, minimizing collateral damage that is usually associated with the use of pesticides that are generally indiscriminate. The findings in this study provide a potentially effective alternative for the development of host plant resistance in bananas against the banana weevil, given the high RNAi efficacy and its species-specific mode of action, adding the RNAi approach to the armory of IPM.

## Figures and Tables

**Table 1:** Components for one liter of artificial diet.

Ingredient	Quantity Per Liter
Agar	20 g
Corm powder	80 g
Dextrose	10 g
Cellulose	14.4 g
Casein	21.6 g
Yeast extract	9.0 g
Wesson’s salt mixture *	2.7 g
Ascorbic acid	1.8 g
Stigmasterol	0.7 g
Nipagin	0.675 g
Potassium sorbet	0.675 g
Inositol	0.36 g
Chlorine chloride	0.45 g
B-vitamin mixture	0.045 g

* Wesson salt is a mixture of eleven different salts used in artificial diets for insects [55,56] and contains all recommended minerals for insect diets.

## Data Availability

The data presented in this study are available on request from the corresponding author.

## Supplementary Materials

The following are available online at https://www.mdpi.com/article/10.3390/insects13010040/s1, Figure S1: cloning strategy: the amplified PCR products of the targets were first TA-cloned into the MCS of the PGEMT-Easy vector (**A**), excised (**B**) with *ApaI* and *Sal1* then subcloned in between the bi-directional T7 promoter of the Litmus38i vectors (**C**) (New England Biolabs, Ipswich, MA, USA); Figure S2: banana weevil eggs incubated on artificial diet and on moist tissue paper (**A**–**C**), larvae on artificial diet (**D**,**E**), adult weevils on artificial diet (**F**–**H**), and pupation, tanning and eclosion on artificial diet (**I**), Figure S3: Top row (**A**–**D**): banana weevil development on artificial diet for 11 days. These larvae burrowed rapidly in the artificial diet. Bottom row: (**E**): 96-well plate with artificial diet with one first-instar larva per well. (**F**): 24-wells plate seeded with 8-day-old larvae and (**H**): 28-day-old larvae transferred to fresh transfer plate; Figure S4: Kaplan–Meier statistical analysis obtained using GraphPad Prism v8.4 (GraphPad, San Diego, CA, USA) compares the mortality of different dsRNA with the dsgfp. Plotted on the same axis, the distance between the curves provides a visual indicator of the relative toxicity of the dsRNA being compared with the control. A, G, and C: dsnf7, dsrps13, and dsmad1 show a high entomotoxicity in Figure S4A,C,G, respectively, while dsvha-A, dsvha-D in Figure S4B,E show intermediate entomotoxicity compared to dslgl and dslaccase2 in Figure S4D,F. Table S1: sequences of target genes in banana weevil (*Cosmopolites sordidus*) used in this study: laccase2, snf7, rps13, mad1, vha-A, vha-D, and lgl; Table S2: primers used in this study; Table S3: statistical analysis to compare weight gain-output from Minitab 19 Statistical Software (State College, PA); Table S4: statistical analysis to compare mortality observed using different dsRNA-output from Minitab 19 Statistical Software (State College, PA, USA); Table S5: computed ET_50_ values at 200 ng of dsRNA per mL of diet; Table S6: cumulative mortality of weevil larvae over a 45 day period.

## References

[1] Gold C.S. (2001). Biology and integrated pest management for the banana weevil *Cosmopolites sordidus* (Germar) (Coleoptera: Curculionidae). Int. J. Pest. Manag..

[2] Gold C.S., Kagezi G.H., Night G., Ragama P. (2004). The effects of banana weevil, *Cosmopolites sordidus*, damage on highland banana growth, yield and stand duration in Uganda. Ann. Appl. Biol..

[3] Gold C., Night G., Abera A., Speijer P. (1998). Hot-water treatment for the control of the banana weevil, *Cosmopolites sordidus* Germar (Coleoptera: Curculionidae), in Uganda. Afr. Entomol..

[4] Udzu A. (1997). Effects of Banana Weevil and Nematode Infestation on the Growth and Yield of Plantain (Mus4. Aab) in Ghana.

[5] Gold C.S., Karamura E.B., Kiggundu A., Bagamba F., Abera A.M.K. (1999). Geographic shifts in the highland cooking banana (*Musa* spp., group AAA-EA) production in Uganda. Int. J. Sust. Dev. World Ecol..

[6] Bosch C., Lorkeers A., Ndile M., Sentozi E. (1996). Diagnostic survey: Constraints to banana productivity in Bukoba and Muleba Districts, Kagera region, Tanzania. Tanzania/Netherlands Farming Systems Research Project. Tanzania. Working paper.

[7] Kema G.H.J., Drenth A., Dita M., Jansen K., Vellema S., Stoorvogel J.J. (2021). Fusarium wilt of banana, a recurring threat to global banana production. Front. Plant. Sci..

[8] Kitavi M., Downing T., Lorenzen J., Karamura D., Onyango M., Nyine M., Ferguson M., Spillane C. (2016). The triploid East African Highland Banana (EAHB) genepool is genetically uniform arising from a single ancestral clone that underwent population expansion by vegetative propagation. Theor. Appl. Genet..

[9] Pearce F. (2008). The sterile banana. Conserv. Manag..

[10] Ehler L.E. (2006). Integrated pest management (IPM): Definition, historical development and implementation, and the other IPM. Pest. Manag. Sci..

[11] Wearing C. (1988). Evaluating the IPM implementation process. Annu. Rev. Entomol..

[12] Shankar U. (2016). Integrated Pest Management in Banana.

[13] Koppenhfer A.M., Reddy K.V.S., Sikora R.A. (1994). Reduction of banana weevil populations with pseudostem traps. Int. J. Pest. Manag..

[14] Masanza M. (2003). Effect of Crop Sanitation on Banana Weevil (*Cosmopolites sordidus*) Populations and Associated Damage. Ph. D. Thesis.

[15] Bryan M.D., Dysart R.J., Burger T.L. (1993). Releases of introduced parasites of the alfalfa weevil in the United States, 1957–1988. Miscellaneous publication/United States Department of Agriculture Animal and Plant Health Inspection Service (USA).

[16] Villani M., Wright R.J. (1988). Entomogenous nematodes as biological control agents of European chafer and Japanese beetle (Coleoptera: Scarabaeidae) larvae infesting turfgrass. J. Econ. Entomol..

[17] Tresson P., Tixier P., Puech W., Carval D. (2021). The challenge of biological control of *Cosmopolites sordidus* Germar (Col. Curculionidae): A review. J. Appl. Entomol..

[18] Waterhouse D., Norris K. (1987). Biological Control: Pacific Prospects.

[19] Roche R., Abreu S. (1983). Control of the banana weevil (*Cosmopolites sordidus*) by the ant *Tetramorium guineense*. Cienc. Agricult..

[20] Castiñeiras A., Ponce E. (1991). Efectividad de la utilización de Pheidole megacephala (Hymenoptera: Formicidae) en la lucha biológica contra Cosmopolites sordidus (Coleoptera: Curculionidae).

[21] Abera M., Gold C.S., Van Driesche R. (2008). Experimental evaluation of the impacts of two ant species on banana weevil in Uganda. Biol. Control..

[22] Tinzaara W., Emudong P., Nankinga C., Tushemereirwe W., Kagezi G., Gold C., Dicke M., Van Huis A., Karamura E. (2015). Enhancing dissemination of Beauveria bassiana with host plant base incision trapfor the management of the banana weevil *Cosmopolites sordidus*. Afr. J. Agricult. Res..

[23] Magara E., Nankinga C., Gold C., Kyamanywa S., Ragama P., Tushemereirwe W., Moore D., Gowen S.R. (2004). Efficacy of *Beauveria bassiana* substrates and formulations for the control of banana weevil. Uganda. J. Agricult. Sci..

[24] Buffington E.J., McDonald S.K. (2006). Banned and Severely Restricted Pesticides.

[25] (2019). PAN International Consolidated List of Banned Pesticides. https://pan-international.org/pan-international-consolidated-list-of-banned-pesticides/.

[26] World Health Organization (1990). Public Health Impact of Pesticides Used in Agriculture.

[27] Collins P.J., Treverrow N.L., Lambkin T.M. (1991). Organophosphorus insecticide resistance and its management in the banana weevil borer, *Cosmopolites sordidus* (Germar) (Coleoptera: Curculionidae), in Australia. Crop. Prot..

[28] Valencia A., Wang H., Soto A., Aristizabal M., Arboleda J.W., Eyun S.-I., Noriega D.D., Siegfried B. (2016). Pyrosequencing the midgut transcriptome of the banana weevil *Cosmopolites Sordidus* (Germar) (Coleoptera: Curculionidae) reveals multiple protease-like transcripts. PLoS ONE.

[29] Gold C.S., Messiaen S. (2000). Musa Pest Fact Sheet on the Banana Weevil Cosmopolites Sordidus.

[30] Kiggundu A., Gold C.S., Labuschagne M.T., Vuylsteke D., Louw S.J.E. (2003). Levels of host plant resistance to banana weevil, *Cosmopolites sordidus* (Germar) (Coleoptera: Curculionidae), in African *Musa* germplasm. Euphytica.

[31] Mansoor S., Amin I., Hussain M., Zafar Y., Briddon R.W. (2006). Engineering novel traits in plants through RNA interference. Trends Plant Sci..

[32] Ding S.-W. (2010). RNA-based antiviral immunity. Nat. Rev. Immunol..

[33] Taning C.N.T., Mezzetti B., Kleter G., Smagghe G., Baraldi E. (2020). Does RNAi-based technology fit within EU sustainability goals?. Trends Biotechnol..

[34] De A., Bose R., Kumar A., Mozumdar S. (2014). Worldwide pesticide use. Targeted Delivery of Pesticides Using Biodegradable Polymeric Nanoparticles.

[35] Rani L., Thapa K., Kanojia N., Sharma N., Singh S., Grewal A.S., Srivastav A.L., Kaushal J. (2021). An extensive review on the consequences of chemical pesticides on human health and environment. J. Cleaner Prod..

[36] Dubelman S., Fischer J., Zapata F., Huizinga K., Jiang C., Uffman J., Levine S., Carson D. (2014). Environmental fate of double-stranded RNA in agricultural soils. PLoS ONE.

[37] Zotti M., Dos Santos E.A., Cagliari D., Christiaens O., Taning C.N.T., Smagghe G. (2018). RNA interference technology in crop protection against arthropod pests, pathogens and nematodes. Pest Manag. Sci..

[38] Taning C.N.T., Gui S., De Schutter K., Jahani M., Castellanos N.L., Christiaens O., Smagghe G. (2021). A sequence complementarity-based approach for evaluating off-target transcript knockdown in *Bombus terrestris*, following ingestion of pest-specific dsRNA. J. Pest Sci..

[39] Ghag S.B., Shekhawat U.K., Ganapathi T.R. (2014). Host-induced post-transcriptional hairpin RNA-mediated gene silencing of vital fungal genes confers efficient resistance against *Fusarium* wilt in banana. Plant Biotechnol. J..

[40] Shekhawat U.K.S., Ganapathi T.R., Hadapad A.B. (2012). Transgenic banana plants expressing small interfering RNAs targeted against viral replication initiation gene display high-level resistance to banana bunchy top virus infection. J. Gen. Virol..

[41] Willow J., Soonvald L., Sulg S., Kaasik R., Silva A.I., Taning C.N.T., Christiaens O., Smagghe G., Veromann E. (2020). First evidence of bud feeding-induced RNAi in a crop pest via exogenous application of dsRNA. Insects.

[42] Laudani F., Strano C.P., Edwards M.G., Malacrinò A., Campolo O., Halim H.M.A.E., Gatehouse A.M., Palmeri V. (2017). RNAi-mediated gene silencing in *Rhynchophorus ferrugineus* (Oliver) (Coleoptera: Curculionidae). Open Life Sci..

[43] Wu K., Taylor C.E., Pinheiro D.H., Skelley L.H., McAuslane H.J., Siegfried B.D. (2019). Lethal RNA interference response in the pepper weevil. J. Appl. Entomol..

[44] Ocimati W., Kiggundu A., Bailey A., Niblett C., Pedun H., Tazuba A. Suppression of the ubiquitin E2 gene through RNA interference causes mortality in the banana weevil, *Cosmopolites sordidus* (Germar). Proceedings of the XXIX International Horticultural Congress on Horticulture: Sustaining Lives, Livelihoods and Landscapes (IHC2014): IX 1114.

[45] Jekayinoluwa T., Tripathi L., Tripathi J.N., Ntui V.O., Obiero G., Muge E., Dale J. (2020). RNAi technology for management of banana bunchy top disease. Food Energy Sec..

[46] Cuillé J. Recherches sur le charancon du bananier (Cosmopolites sordidus, Germ: SDTC; 1950. https://agritrop.cirad.fr/471368/1/ID471368.pdf.

[47] Beccari F. (1967). Contributo alla conoseenza del *Cosmopolites sordidus* Ger. (Coleoptera: Curculionidae), parte I-II. Rev. Agric. Subtrop..

[48] Koppenhöfer A. (1993). Observations on egg-laying behaviour of the banana weevil, *Cosmopolites sordidus* (Germar). Entomol. Exp. Appl..

[49] Levy-Booth D.J., Campbell R.G., Gulden R.H., Hart M.M., Powell J.R., Klironomos J.N., Pauls K.P., Swanton C.J., Trevors J.T., Dunfield K.E. (2007). Cycling of extracellular DNA in the soil environment. Soil Biol. Biochem..

[50] Blum S.A., Lorenz M.G., Wackernagel W. (1997). Mechanism of retarded DNA degradation and prokaryotic origin of DNases in nonsterile soils. Syst. Appl. Microb..

[51] Bakaze E., Kiggundu A., Tushemereirwe W. (2018). Use of artificial diets with plant material to evaluate banana cultivars for resistance to *Cosmopolites sordidus*. Uganda J. Agric. Sci..

[52] Prentice K., Pertry I., Christiaens O., Bauters L., Bailey A., Niblett C., Ghislain M., Gheysen G., Smagghe G. (2015). Transcriptome analysis and systemic RNAi response in the African sweet potato weevil (*Cylas puncticollis*, Coleoptera, Brentidae). PLoS ONE.

[53] Longoria A.G.G. (1968). Diferencias Sexuales en la Morfologia Externa de Cosmopolites Sordidus Germar (Coleoptera, Curculionidae).

[54] Ekobu M., Solera M., Kyamanywa S., Mwanga R.O., Odongo B., Ghislain M., Moar W.J. (2010). Toxicity of seven *Bacillus thuringiensis* Cry proteins against *Cylas puncticollis* and *Cylas brunneus* (Coleoptera: Brentidae) using a novel artificial diet. J. Econ. Entomol..

[55] Wang Y., Zheng Z., Zhou Y. (1986). Book of Insect Artificial Food.

[56] Willis R., Allen P. (1999). Measurement of amorphous ferric phosphate to assess iron bioavailability in diets and diet ingredients. Analyst.

[57] Mezzetti B., Smagghe G., Arpaia S., Christiaens O., Dietz-Pfeilstetter A., Jones H., Kostov K., Sabbadini S., Opsahl-Sorteberg H.G., Ventura V. (2020). RNAi: What is its position in agriculture?. J. Pest. Sci..

[58] Christiaens O., Prentice K., Pertry I., Ghislain M., Bailey A., Niblett C., Gheysen G., Smagghe G. (2016). RNA interference: A promising biopesticide strategy against the African sweet potato weevil *Cylas brunneus*. Sci. Rep..

[59] Baum J.A., Bogaert T., Clinton W., Heck G.R., Feldmann P., Ilagan O., Johnson S., Plaetinck G., Munyikwa T., Pleau M. (2007). Control of coleopteran insect pests through RNA interference. Nat. Biotech..

[60] Bolognesi R., Ramaseshadri P., Anderson J., Bachman P., Clinton W., Flannagan R., Ilagan O., Lawrence C., Levine S., Moar W. (2012). Characterizing the mechanism of action of double-stranded RNA activity against western corn rootworm (*Diabrotica virgifera virgifera* LeConte). PLoS ONE.

[61] Li H., Khajuria C., Rangasamy M., Gandra P., Fitter M., Geng C., Woosely A., Hasler J., Schulenberg G., Worden S. (2015). Long dsRNA but not siRNA initiates RNAi in western corn rootworm larvae and adults. J. Appl. Entomol..

[62] Arakane Y., Muthukrishnan S., Beeman R.W., Kanost M.R., Kramer K.J. (2005). Laccase 2 is the phenoloxidase gene required for beetle cuticle tanning. Proc. Natl. Acad. Sci. USA.

[63] Prentice K., Christiaens O., Pertry I., Bailey A., Niblett C., Ghislain M., Gheysen G., Smagghe G. (2017). RNAi-based gene silencing through dsRNA injection or ingestion against the African sweet potato weevil *Cylas puncticollis* (Coleoptera: Brentidae). Pest Manag. Sci..

[64] Sharma R., Christiaens O., Taning C.N.T., Smagghe G. (2021). RNAi-mediated mortality in southern green stinkbug *Nezara viridula* by oral delivery of dsRNA. Pest Manag. Sci..

[65] Heinrich S., Sewart K., Windecker H., Langegger M., Schmidt N., Hustedt N., Hauf S. (2014). Mad1 contribution to spindle assembly checkpoint signalling goes beyond presenting Mad2 at kinetochores. EMBO Rep..

[66] Emre D., Terracol R., Poncet A., Rahmani Z., Karess R.E. (2011). A mitotic role for Mad1 beyond the spindle checkpoint. J. Cell. Sci..

[67] Forgac M. (2007). Vacuolar ATPases: Rotary proton pumps in physiology and pathophysiology. Nat. Rev. Mol. Cell. Biol..

[68] Mao J., Zhang P., Liu C., Zeng F. (2015). Co-silence of the coatomer beta and v-ATPase A genes by siRNA feeding reduces larval survival rate and weight gain of cotton bollworm, *Helicoverpa armigera*. Pestic. Biochem. Physiol..

[69] Xiao D., Liang X., Gao X., Yao J., Zhu K.Y. (2014). The lethal giant larvae gene in *Tribolium castaneum*: Molecular properties and roles in larval and pupal development as revealed by RNA interference. Int. J. Mol. Sci..

[70] Yuan B., Latek R., Hossbach M., Tuschl T., Lewitter F. (2004). siRNA Selection Server: An automated siRNA oligonucleotide prediction server. Nucleic Acids Res..

[71] Sarathi M., Simon M.C., Ahmed V.I., Kumar S.R., Hameed A.S. (2008). Silencing VP28 gene of white spot syndrome virus of shrimp by bacterially expressed dsRNA. Marine Biotech..

[72] Taning C.N.T., Christiaens O., Berkvens N., Casteels H., Maes M., Smagghe G. (2016). Oral RNAi to control *Drosophila suzukii*: Laboratory testing against larval and adult stages. J. Pest Sci..

[73] Nwokeoji A.O., Kilby P.M., Portwood D.E., Dickman M.J. (2017). Accurate quantification of nucleic acids using hypochromicity measurements in conjunction with UV spectrophotometry. Anal. Chem..

[74] Night G., Gold C., Power A. (2011). Feeding behaviour and efficiency of banana weevil (*Cosmopolites sordidus*) larvae on banana cultivars of varying resistance levels. J. Appl. Entomol..

[75] Pinheiro D.H., Taylor C.E., Wu K., Siegfried B.D. (2020). Delivery of gene-specific dsRNA by microinjection and feeding induces RNAi response in Sri Lanka weevil, *Myllocerus undecimpustulatus undatus* Marshall. Pest Manag. Sci..

[76] Christiaens O., Whyard S., Vélez A.M., Smagghe G. (2020). Double-stranded RNA technology to control insect pests: Current status and challenges. Front. Plant Sci..

[77] Ramaseshadri P., Segers G., Flannagan R., Wiggins E., Clinton W., Ilagan O., McNulty B., Clark T., Bolognesi R. (2013). Physiological and cellular responses caused by RNAi-mediated suppression of Snf7 orthologue in western corn rootworm (*Diabrotica virgifera virgifera*) larvae. PLoS ONE.

[78] Wu K., Taylor C.E., Fishilevich E., Narva K.E., Siegfried B.D. (2018). Rapid and persistent RNAi response in western corn rootworm adults. Pest. Biochem. Physiol..

[79] Bachman P.M., Bolognesi R., Moar W.J., Mueller G.M., Paradise M.S., Ramaseshadri P., Tan J., Uffman J.P., Warren J., Wiggins B.E. (2013). Characterization of the spectrum of insecticidal activity of a double-stranded RNA with targeted activity against western corn rootworm (*Diabrotica virgifera virgifera* LeConte). Transgenic Res..

[80] Christiaens O., Sweet J., Dzhambazova T., Urru I., Smagghe G., Kostov K., Arpaia S. (2021). Implementation of RNAi-based arthropod pest control: Environmental risks, potential for resistance and regulatory considerations. J. Pest Sci..

[81] De Schutter K., Taning C.N.T., Van Daele L., Van Damme E.J.M., Dubruel P., Smagghe G. (2021). RNAi-based biocontrol products: Market status, regulatory aspects and risk assessment. Front. Insect Sci..

[82] Huvenne H., Smagghe G. (2010). Mechanisms of dsRNA uptake in insects and potential of RNAi for pest control: A review. J. Insect Physiol..

[83] Niu Q.-W., Lin S.-S., Reyes J.L., Chen K.-C., Wu H.-W., Yeh S.-D., Chua N.H. (2006). Expression of artificial microRNAs in transgenic *Arabidopsis thaliana* confers virus resistance. Nat. Biotech..

[84] Nunes C.C., Dean R.A. (2012). Host-induced gene silencing: A tool for understanding fungal host interaction and for developing novel disease control strategies. Mol. Plant Pathol..

[85] Yoder J.I., Gunathilake P., Wu B., Tomilova N., Tomilov A.A. (2009). Engineering host resistance against parasitic weeds with RNA interference. Pest Manag. Sci..

