# Supplementary material for: First Evidence of Feeding-Induced RNAi in Banana Weevil via Exogenous Application of dsRNA
Source: Insects. 2021 Dec 29;13(1):40. doi: 10.3390/insects13010040 (PMC8779063; doi:10.3390/insects13010040)
Supplement: Supplementary file 1 [file insects-13-00040-s001.zip › insects-1493736-supplementary.pdf]

insects

MDPI

Supplementary Materials

First Evidence of Feeding-Induced RNAi in Banana Weevil via Exogenous Application of dsRNA

Henry Shaykins Mwaka, Olivier Christiaens, Priver Namanya Bwesigye, Jerome Kubiriba, Wilberforce Kateera Tushemereirwe, Godelieve Gheysen and Guy Smagghe

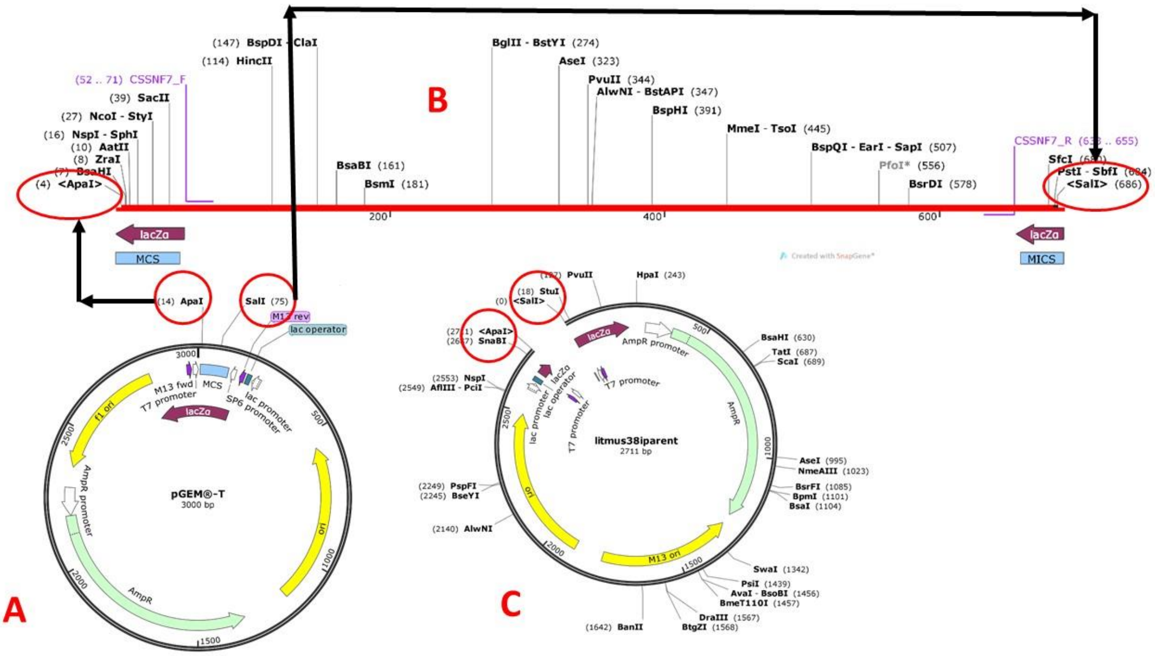

Figure S1. cloning strategy: the amplified PCR products of the targets were first TA-cloned into the MCS of the PGEMT-Easy vector (**A**), excised (**B**) with *ApaI* and *Sal1* then subcloned in between the bi-directional T7 promoter of the Litmus38i vectors (**C**) (New England Biolabs, Ipswich, MA, USA).

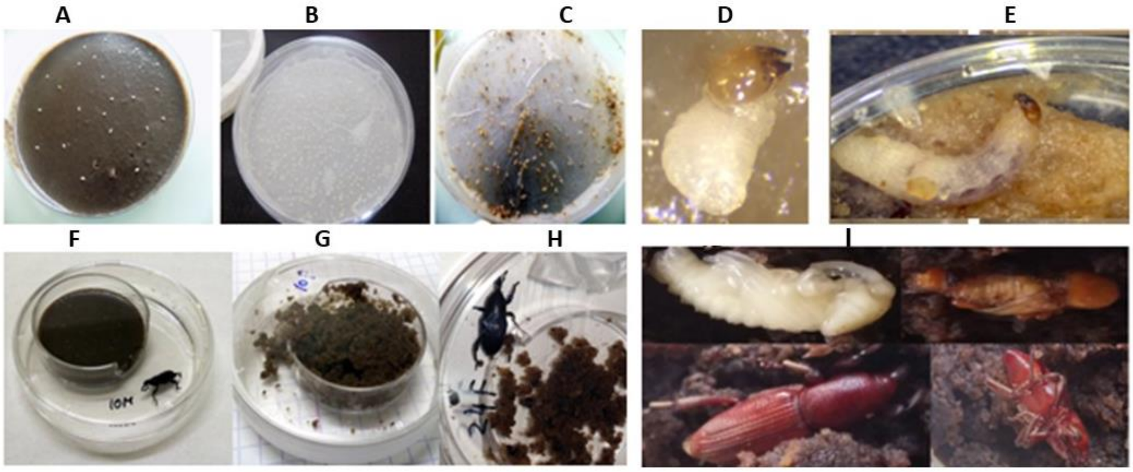

Figure S2. Banana weevil eggs incubated on artificial diet and on moist tissue paper (**A**–**C**), larvae on artificial diet (**D**, **E**), adult weevils on artificial diet (**F**–**H**), and pupation, tanning and eclosion on artificial diet (**I**).

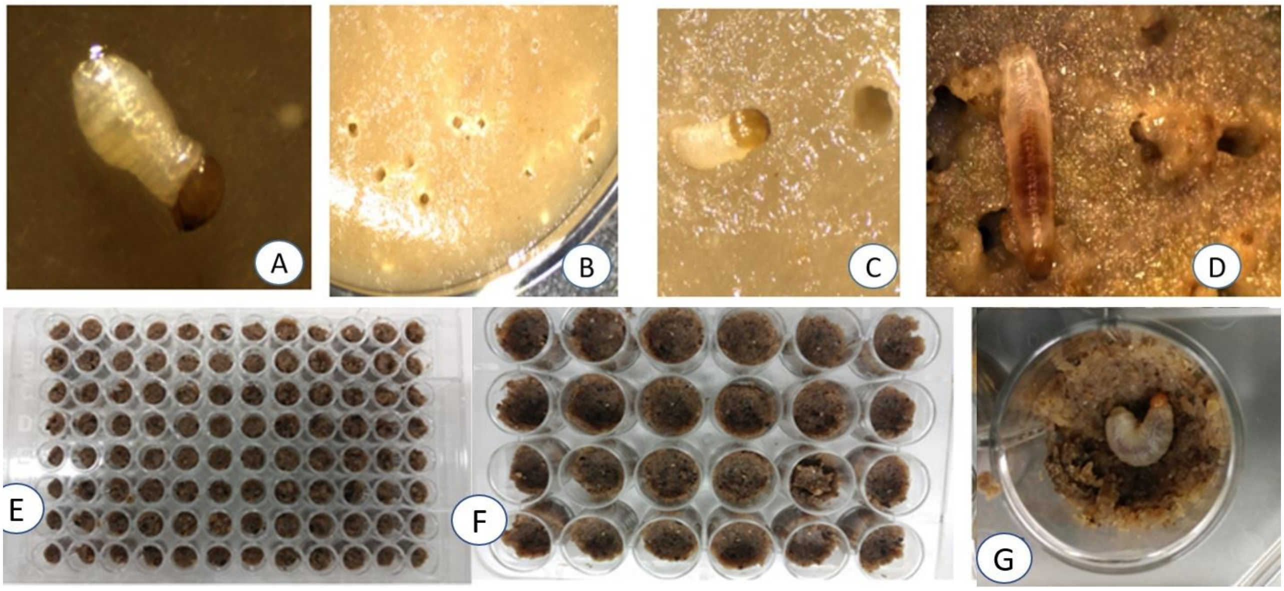

Figure S3. Top row (**A–D**): banana weevil development on artificial diet for 11 days. These larvae burrowed rapidly in the artificial diet. Bottom row: E: 96-well plate with artificial diet with one first-instar larva per well. F: 24-wells plate seeded with 8-day-old larvae and H: 28-day-old larvae transferred to fresh transfer plate.

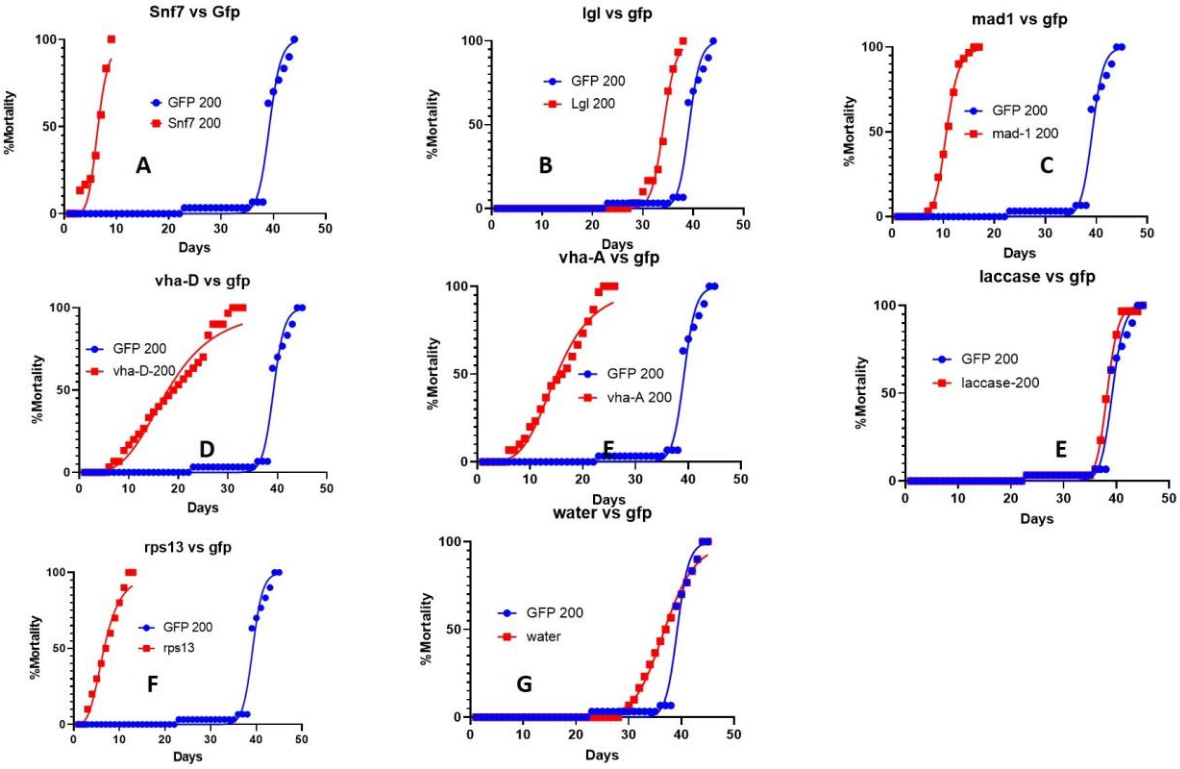

Figure S4. Kaplan–Meier statistical analysis obtained using GraphPad Prism v8.4 (GraphPad, San Diego, CA, USA) compares the mortality of different dsRNA with the dsgfp. Plotted on the same axis, the distance between the curves provides a visual indicator of the relative toxicity of the dsRNA being compared with the control. A, G, and C: dsnf7, dsrps13, and dsmad1 show a high entomotoxicity in Figure S4A, G and C, respectively, while dsvha-A, dsvha-D in Figure S4B and E show intermediate entomotoxicity compared to dslgl and dslaccase2 in Figure S4D and F.

Table S1. Sequences of target genes in banana weevil (*Cosmopolites sordidus*) used in this study: laccase2, snf7, rps13, mad1, vha-A, vha-D, and lgl.

>laccase2

CATCTAAATTGATAAAAGAAGAAAAATTTACTAGAAGATTTGATACGTCTCTGTGTAAATCAGAATTAGGAAAAGTCTGT CTTGGAGACATAAAATCCCTAGAAAAACTCCCAGAAGAACTGAAAGGAAATGAAGTGGATCGAAAAATATACTTAGACA TCGATTTTAAAACCACCGGGAGAAATTCAAACGTAAACGGTGGATTTACGGATCTCCGTAGCAAAATATATAGGATAAAC AATCTGACTTTTGCCTATCCCTCTTCACCTCTTCTTACCCAACCGATGGATGTGCCATCTGCTTTAATTTGCAACGAAATGAT GGTGCCGGGAGTATGCCAGACAACCAACATCTGCGAGT
**>snf7**
ATGACAACTATCTGACATATACCTGTAAAAGAGAAAAATTGCGTTATTTGTTGGAGTTGGTGACTCATAGTGTGCTCATAG TTATTTTTTGCAACAATATAAAATTTGCAATATTAATTTAGGCCTTAAGCATGTCATTTTTCGGTAAAATATTCGGTGGAAA AAAAGATGAAGCCCCGTCCACTAGTGACGCTATCCAAAAATTAAGGGAAACACAAGACTTGTTGACGAAAAAACAGGAG TATTTAGAGAAACAAATCGATGATTTGATTCTAATCGCCAAAAAGAATGCTTCAAAAAATAAGCGAGTTGCTTTGCAAGC ATTGAAAAAGAAGAAAAGATTGGAAAAGACATTACAACAAGTAGATGGGACTTTGACAACATTAGATCTCCAAAAGGAT GCTTTAGAAGGAGCTAACACCAATGCTGCTGTATTAATTTCAATGAGAGATGCAGCTGCTGCTTTGAAAAATGCCCATAAG AACCTCGACATAGACAATGTTCATGATATAATGGATGATATTTGCTGAACAACATGATTTAGCCAATGAAATATCCAATGCT ATTAGCAATCCAGTCGGATTTGGTGATGATATTGATGAGGATGAATTAAACAAAGAATTGGAAGAGCTTGAAACTGAGAA CTTTGAATCAGACCTTATTGATGTTCCTGGACCAACTAAACTGCCTCCATTGCCTACCAAAATTGGTGAAGAAGAACCAGC TAAAATCAAAAAGCCAGCAAAGAAAGTCGAAGAAGATGAGGACTAT
**>rps13**
AACCTGACATGCGTAATACGAGAATAATGGCAGCTCGTAGAAAATTTGTTGACAAGTAATTCTTTTTAGCGTAGCGGTCCT TTTCTTGTACGCGAATCAAGTAAAAATTGATAAAAAATGGGTCGTATGCACGCACCTGGAAAAGGTATTGCCCAGTCGGC TTTACCTTATAGAAGAAGTGTACCAACATGGCTCAAAGTAACGCCAGAGGAAGTAAAAGAGCATATTGTCAAACTTGGCA AGAAGGGTCTTACTCCCTCACAAATTGGTGTTATCCTTAGGGATTCGTATGGCGTTGCCCAAGTACGTTTTGTAACAGGTAA TAAGATACTTAGAATTATGAAAGCAGTGGGCCTAGCTCCTGATCTACCTGAAGATTTGTATTACTTGATCAAAAAAGCTGT AGCTATTCGTAAACATTTAGAAAGAAACAGGAAGGATAAGGACAGCAAATTCCGTCTTATTCTGGTTGAGTCCCGTATTC ACCGCTTAGCTAGATATTACAAGACTAAAAATGTCCTAGCACCCAACTGGAAATATGAATCCAGCACAGCATCAGCTCTG GTTGCTTAATATTATGAAATTTTGTTATATATAAGCGAAAAATAAATATAAAAATGTCCT
**>mad1**
CCTTTTGAGATCTTCTATTGAGAAATTACAACAGCAAGAAATTGGATTGATGTCTGAGAAAGTTGAGTTTGAGTCCCAGTTT AGAGCTGCTGAACATGCTGCCAAAGTAGCAAGATCAGAACTAGAAAAGAACCAAAAATTACTGAGCAACTTGAAAACA ACAGGTGAACAAAAACAATTATTGATTCATCGCATGCAAAAAAAACTTCTTCTTGTTAGCCGTGAGAGAGACAGTTACAG ATTACAATTAGATTCATATGAGAGAGATCTTACTATGGTTGATAATAGTTCACATGAAGCAACACCTGGTTCAAAAGTAAC ACATAGTTACAAAGAGAGGATTGACAACTTGGAAAAAGTTGTTGATGGTTATAGAGATATGGTGGCCAAGTTAGAGAGTG ATCTTCAGTTGACACAACCACAACTATATACAGATGTGACACCGGTAAGAGCAGAACAAATAACAAGACTTCAAGATGA TGCAGCCAAGTTAAGGGAAGAAAACAAAATGTTGAGGGAACAAAAAGATAAATTAGAAATCAAACTGGAATCATTGCTA GAAGGTCAGGATACTTTCCATGGTGGGCAGATTGTACATCTAGCAAACAATCCTCTTTCACAGTGCACTGCAGTAAAAGCC AATCTTATCGACCAGTTACAACAAGAAAACTTAAGATTGAAAAACAAACTCAAGAAAATGGAAGAAGGCATAGAAACC AGCGTCTGGGGGGATATTTCAATATGTTCAAAAGAAGTTCAAGTCCTGCGAGAGCAAATAAAAAATAACGAAAAACAAA GTCAGAGACTCAAAGACTATTTCAAATCGTCCATGCAGGACTTTAGAAATGTAATTTACATGCTGTTTGGTTACAAGATAG ATAAACCTTCTAACTCTAGCATTTACAAACTGAGAAGCATGTACGCGTTGCATGCAGAGGATCAGCTTTGTTTTGAAGTTA ATCCTGAGGGTGACTTAAATCTGTTGGAAAATGAGTTCAGCGCCACCTTGGGGCCCATGATAGATTTACATTTGATTCATC AAAATTCGATTCCAGTTTTCTTGAGCGGCATCACTATGGACTTGTTTAATCAGAAGACAATGACCAAAGTATATTAACGAC ATTTTGATTCAGTCAGTTGTATTTTAAGTTGCTGTCAATGTAGAAGCATATTTTAATGATATAGTATGAACATTTTAATATTA CATATAATAAA

>vha-A

TTGGCCTCCTTCTACGAACGTGCTGGTCGCGTTAAGTGTTTGGGTAATCCCGACAGAGAAGGTTCGGTCTCCATTGTCGGTG
CTGTATCACCCCCTGGTGGTGACTTCTCCGATCCCGTCACTTCAGCCACTCTGGGTATTGTACAGGTGTTTTGGGGTTTGGA
CAAAAAACTGGCTCAACGTAAGCACTTCCCTTCGGTCAACTGGTTGATTTCTTATTCCAAATACATGCGTGCCCTTGATGAC
TTCTACGACAAGAACTTCGCCGAATTCGTGCCCCTCAGAACTAAAGCCAAGGAAATTCTTCAGGAAGAAGAAGATTTGTC
CGAAATCGTGCAGCTGGTAGGTAAGGCTTCCCTGGCCGAAACCGACAAGATCACCTTGGAAGTAGCCAAGTTACTCAAGG
AGGATTTCTTGCAACAGAACTCTTATTCTGCGTACGATCGATTCTGTCCATTCTACAAAACAGTTGGTATGCTTAAGAACAT
GATCGGTCTCTACGATATGGCCAGACACGCCGTCGAAAGTACCGCACAATCCGAGAACAAGATCACCTGGTCCGTCATCA
GAGATTCCATGAGCAATATCCTCTACCAACT

>vha-D

GTTAATGAAAGGTCGTCTTAAGGGAGCACAAAAGGGCCACAGTTTGCTGAAAAAGAAAGCAGATGCTCTACAAATGCGT
TTCCGTATGATTTTAAGTAAAATTATCGAGACAAAAACTTTAATGGGGGATGTGATGAAGGAGGCAGCTTTTTCATTGGCG
GAAGCAAAGTTTACTACAGGCGACTTCAATCAAGTTGTTTTACAGAATGTAACTAAGGCTCAACTGAAAATCCGCACCAA
AAAAGATAATGTAGCTGGTGTTACCTTGCCCGTGTTTGAGTGGTATCAAGATGGAACAGACAATTATGAACTGGCCGGTTT
AGCTAGAGGTGGTCAACAGTTGGCCAAACTGAAGAAGAATTATCAAAGTGCTATAAAATTGTTGGTAGAATTGGCATCTC
TACAAACATCTTTTGTCACCTTAGATGAAGTAATTAAAATCACGAACAGAAGAGTGAATGCCATTGAACATGTTATTATCC
CTAAAATTGAGAACACCCTCGCCTATATTATTTCCGAACTGGATGAAATTGAAAGAGAAGAGTTCTACCGTTTAAAGAAG
ATTCAAGATAAAAAGAAGATCAACAAAGCCAAGGCAGAAAAGGCCAAAGCTGAG

>lgl

TTTTAGACGATTATGCCCGAACGTTCCTTGTTAATGTACAAAATACCATACCAACCCTTTGGGCTGGTACCAACAATGGCA
CAGTTTACGCATTTACAATAATGGTTCCATCAGCATCGAAGAGAGACACAGATGATGTAGCCTGCCACCTAGCCAAAGAG
ATACAATTAAAACACAGAGCACCTGTAATTGGAATTGCTGTACTGGATGGATCCAGCAAACCGCTACCAGAACCACTTGA
AGTGGAGAAAGGCGTGGCACCTCTTCCCGATACAACGCAAGCGCACAGAGTCGTCATAGCATCCGAGGAACAATTCAAA
ATATTTACGTTGCCATCACTAAAACCATATTGTAAATTTAAGCTTACCGCACACGAAGGAGCTAGAGTTCGAAGGATGGCT
TTTGCTACATTCTCTTGCTTAATGCCAGACGACAATACAAAATATTCAGAAGTGGATCTTTTGTGCCTTACCAATATGGGTG
ATTGCCTCATACTGACTATTCCAGATTTAAAACGTCAATTAAATTCGGCAGCTATAAAACGGGAAGATATCAATGGAATAT
CTTCTCTCGTGTTCACAAAGCAAGCTGAAGCACTGTACCTCCATTCCTCATCGGAATTGCAGAGGATATCATTGTCAGCCA
CTGCAATAACTCAGGCTAGGTGTTACCTTCCACTACCAGCAAAGGAAGACGGTGTTTCCGAATCAGGA

Key: Yellow highlighted region is the dsRNA target region used in this study. Blue highlighted region is the position of primers for cloning dsRNA. Green highlighted and underlined region is the position of primers used for expression analysis

Table 2. Primers used in this study.

Gene name	Primer name	Forward	Reverse	Purpose	product size
laccase2	Cs-S-lac	CATCTAAATTGATAAAAG	GCAGATGTTGGTTGTCTGGCAT	dsRNA production	356
snf7	Cs-S-SNF7	AAAAGATGAAGCCCCGTCCA	GTCCTCATCTTCTTCGACTTTCT	dsRNA production	604
snf7	Cs-snf7-sq	TGCGTTATTTGTTGGAGTTGGT	TGGCTAAATCATGTTGTTCAGCA	RT-PCR	507
rps13	C-RPS13	GTATGCACGCACCTGGAAAA	CCAGTTGGGTGCTAGGACAT	dsRNA production	410
rps13	Cs-S-RPS13-sq	TCCTTTTCTTGTACGCGAATCAA	CTGATGCTGTGCTGGATTCATAT	RT-PCR	482
mad1	Cs-MAD1	ATGTGACACCGGTAAGAGCA	CAACGCGTACATGCTTCTCA	dsRNA production	494

mad1	Cs-S-mad1-sq	GCCAAAGTAGCAAGATCAGAACT	TGCACTGTGAAAGAGGATTGTTT	RT-PCR	526
vha-A	Cs-S-vATPaseA	GTCGCGTTAAGTGTTTGGGT	GTTCTCGGATTGTGCGGTAC	dsRNA production	521
vha-D	Cs-S-vATPaseD	AGGTCGTCTTAAGGGAGCAC	TTCTGCCTTGGCTTTGTTGA	dsRNA production	592
lgl	Cs-S-LGL	TGATGTAGCCTGCCACCTAG	ACCGTCTTCCTTTGCTGGTA	dsRNA production	565
gfp	S-gfp	TACGGCGTGCAGTGCT	TGATCGCGCTTCTCG	dsRNA production	455
β-actin	Cs-act-sq	AAGACATCAGGGCGTAATGG	GAAGGTGTGGTGCCAGATTT	RT-PCR	200

Table S3. statistical analysis to compare weight gain-output from Minitab 19 Statistical Software (State College, PA).

One-way ANOVA: Weight in mg versus age in days

A Factor Information

Factor	Levels	Values
age in days	17	4, 8, 12, 16, 20, 24, 28, 32, 36, 40, 44, 48, 52, 56, 60, 64, 68

B Means

age in days	N	Mean	StDev	95% CI
4	10	11.700	1.767	(9.479, 13.921)
8	10	18.600	2.633	(16.379, 20.821)
12	10	25.30	3.56	(23.08, 27.52)
16	10	38.200	3.084	(35.979, 40.421)
20	10	47.900	1.792	(45.679, 50.121)
24	10	66.60	3.44	(64.38, 68.82)
28	10	90.000	3.018	(87.779, 92.221)
32	10	107.00	3.46	(104.78, 109.22)
36	10	118.300	3.057	(116.079, 120.521)
40	10	137.100	2.685	(134.879, 139.321)
44	10	143.500	2.759	(141.279, 145.721)
48	10	148.70	4.69	(146.48, 150.92)
52	10	140.30	5.12	(138.08, 142.52)
56	9	129.78	4.32	(127.44, 132.12)
60	10	115.20	4.13	(112.98, 117.42)
64	10	94.60	4.55	(92.38, 96.82)
68	10	84.70	4.27	(82.48, 86.92)

Pooled StDev = 3.55489

C Grouping Information Using the Fisher LSD Method and 95% Confidence

age in days	N	Mean	Grouping
48	10	148.70	A
44	10	143.500	B
52	10	140.30	C
40	10	137.100	D
56	9	129.78	E
36	10	118.300	F
60	10	115.20	F
32	10	107.00	G
64	10	94.60	H
28	10	90.000	I
68	10	84.70	J
24	10	66.60	K
20	10	47.900	L
16	10	38.200	M
12	10	25.30	N
8	10	18.600	O
4	10	11.700	P

Means that do not share a letter are significantly different.

D Statistics **Days to pupation**

Variable	N	N*	Mean	SE Mean	StDev	Minimum	Q1	Median	Q3	Maximum
Days to pupation	10	0	53.40	1.49	4.72	44.00	51.00	54.00	56.50	60.00

Table S3: A-E Statistical analysis to compare weight with age-output from Minitab 19 Statistical Software (State College, PA)

Table S4. Statistical analysis to compare mortality observed using different dsRNA-output from Minitab 19 Statistical Software (State College, PA, USA).

A Factor Information

Factor	Levels	Values
Factor	8	water, gfp, snf7, vha-A, rps13, vha-D, mad-1, lgl

B Analysis of Variance

Source	DF	Adj SS	Adj MS	F-Value	P-Value
Factor	7	38973	5567.64	317.80	0.000
Error	232	4065	17.52		
Total	239	43038			

C Model Summary

S	R-sq	R-sq(adj)	R-sq(pred)
4.18563	90.56%	90.27%	89.89%

D Means

Factor	N	Mean	StDev	95% CI
water	30	36.400	4.484	(34.894, 37.906)
gfp	30	38.533	3.530	(37.028, 40.039)
snf7	30	5.767	1.924	(4.261, 7.272)
vha-A	30	14.967	5.449	(13.461, 16.472)
rps13	30	6.500	2.921	(4.994, 8.006)
vha-D	30	18.13	7.48	(16.63, 19.64)
mad-1	30	10.233	1.977	(8.728, 11.739)
lgl	30	33.400	2.401	(31.894, 34.906)

Pooled StDev = 4.18563

E Grouping Information Using the Tukey Method and 95% Confidence

Factor	N	Mean	Grouping
gfp	30	38.533	A
water	30	36.400	A B
lgl	30	33.400	B
vha-D	30	18.13	C
vha-A	30	14.967	C
mad-1	30	10.233	D
rps13	30	6.500	E
snf7	30	5.767	E

Means that do not share a letter are significantly different.

Table S4: A-E Statistical analysis to compare mortality observed using different dsRNA-output from Minitab 19 Statistical Software (State College, PA)

Table S5. Computed ET_{50} values at 200 ng of dsRNA per mL of diet.

[dsRNA] vs. normalized response -- Variable slope	gfp	snf7	lgl	mad1	vha-D	vha-A	rps13	Water
Best-fit values								
HillSlope	35.76	6.346	28.16	8.907	3.614	4.274	3.555	12.57
ET50	39.22	6.448	34.15	10.62	17.9	15.1	6.642	36.82
logET50	1.594	0.8094	1.533	1.026	1.253	1.179	0.8223	1.566
Std. Error								
HillSlope	3.561	1.265	1.623	0.3919	0.2421	0.3161	0.3474	0.4083
ET50	0.1237	0.2134	0.07787	0.05985	0.3407	0.2733	0.1948	0.1022
95% CI (profile likelihood)								
HillSlope	28.81 to 46.08	3.682 to 11.52	24.79 to 32.26	8.145 to 9.776	3.157 to 4.146	3.678 to 4.981	2.880 to 4.419	11.82 to 13.38
ET50	38.98 to 39.48	5.870 to 6.968	33.98 to 34.31	10.49 to 10.75	17.17 to 18.62	14.51 to 15.68	6.192 to 7.088	36.61 to 37.03
logET50	1.591 to 1.596	0.7686 to 0.8431	1.531 to 1.535	1.021 to 1.031	1.235 to 1.270	1.162 to 1.195	0.7918 to 0.8505	1.564 to 1.569
Goodness of Fit								
Degrees of Freedom	42	7	36	15	31	24	11	43
R squared	0.9707	0.9515	0.9892	0.9975	0.9708	0.9752	0.9778	0.9943
Sum of Squares	975.8	509.9	299	74.49	1151	813.4	354.8	252.1
Sy.x	4.82	8.535	2.882	2.228	6.094	5.822	5.68	2.422
Number of points								
# of X values	44	9	38	17	33	26	13	45
# Y values analyzed	44	9	38	17	33	26	13	45

Table S5: ET50 values at 200ng of dsRNA per ml of diet-computed with Prism v8.4 (GraphPad, San Diego, CA)

Table 6. Cumulative mortality of weevil larvae over a 45 day period.

Days	dsgfp	dssnf7	dsrps13	dsmad1	dsvha-A	dsvha-D	dslgl	water
1	0.00	0.00	0.00	0.00	0.00	0.00	0.00	0.00
2	0.00	0.00	0.00	0.00	0.00	0.00	0.00	0.00
3	0.00	13.33	10.00	0.00	0.00	0.00	0.00	0.00
4	0.00	16.67	20.00	0.00	0.00	0.00	0.00	0.00
5	0.00	20.00	30.00	0.00	0.00	0.00	0.00	0.00
6	0.00	33.33	40.00	0.00	6.67	3.33	0.00	0.00
7	0.00	56.67	50.00	3.33	6.67	6.67	0.00	0.00
8	0.00	83.33	60.00	6.67	10.00	6.67	0.00	0.00
9	0.00	100.00	70.00	23.33	13.33	13.33	0.00	0.00
10	0.00		80.00	36.67	20.00	16.67	0.00	0.00
11	0.00		90.00	53.33	23.33	20.00	0.00	0.00
12	0.00		100.00	73.33	30.00	23.33	0.00	0.00
13	0.00			90.00	36.67	26.67	0.00	0.00
14	0.00			93.33	43.33	33.33	0.00	0.00
15	0.00			96.67	46.67	36.67	0.00	0.00
16	0.00			100.00	50.00	40.00	0.00	0.00
17	0.00				53.33	43.33	0.00	0.00
18	0.00				60.00	46.67	0.00	0.00
19	0.00				66.67	50.00	0.00	0.00
20	0.00				73.33	53.33	0.00	0.00
21	0.00				80.00	56.67	0.00	0.00
22	0.00				86.67	60.00	0.00	0.00
23	3.33				96.67	63.33	0.00	0.00
24	3.33				100.00	66.67	0.00	0.00
25	3.33					70.00	0.00	0.00
26	3.33					83.33	0.00	0.00
27	3.33					90.00	0.00	0.00
28	3.33					90.00	3.33	0.00
29	3.33					90.00	3.33	3.33
30	3.33					96.67	10.00	6.67
31	3.33					100.00	16.67	10.00
32	3.33						16.67	16.67
33	3.33						23.33	23.33
34	3.33						40.00	30.00
35	3.33						70.00	36.67
36	6.67						83.33	43.33
37	6.67						93.33	50.00
38	6.67						100.00	56.67
39	63.33							63.33
40	70.00							70.00
41	76.67							76.67
42	83.33							83.33
43	90.00							90.00
44	100.00							100.00
45								

Table S6: Cumulative mortality of weevil larvae over a 45 day period
